# Functional changes of default mode network and structural alterations of gray matter in patients with irritable bowel syndrome: a meta-analysis of whole-brain studies

**DOI:** 10.3389/fnins.2023.1236069

**Published:** 2023-10-24

**Authors:** Mengqi Zhao, Zeqi Hao, Mengting Li, Hongyu Xi, Su Hu, Jianjie Wen, Yanyan Gao, Collins Opoku Antwi, Xize Jia, Yang Yu, Jun Ren

**Affiliations:** ^1^School of Psychology, Zhejiang Normal University, Jinhua, China; ^2^Key Laboratory of Intelligent, Education Technology and Application of Zhejiang Province, Zhejiang Normal University, Jinhua, China; ^3^School of Western Languages, Heilongjiang University, Harbin, China; ^4^Department of Psychiatry, The Second Affiliated Hospital, School of Medicine, Zhejiang University, Hangzhou, China

**Keywords:** irritable bowel syndrome, meta-analysis, default mode network, functional connectivity, voxel-based morphometry

## Abstract

**Background:**

Irritable bowel syndrome (IBS) is a brain-gut disorder with high global prevalence, resulting from abnormalities in brain connectivity of the default mode network and aberrant changes in gray matter (GM). However, the findings of previous studies about IBS were divergent. Therefore, we conducted a meta-analysis to identify common functional and structural alterations in IBS patients.

**Methods:**

Altogether, we identified 12 studies involving 194 IBS patients and 230 healthy controls (HCs) from six databases using whole-brain resting state functional connectivity (rs-FC) and voxel-based morphometry. Anisotropic effect-size signed differential mapping (AES-SDM) was used to identify abnormal functional and structural changes as well as the overlap brain regions between dysconnectivity and GM alterations.

**Results:**

Findings indicated that, compared with HCs, IBS patients showed abnormal rs-FC in left inferior parietal gyrus, left lingual gyrus, right angular gyrus, right precuneus, right amygdala, right median cingulate cortex, and left hippocampus. Altered GM was detected in the fusiform gyrus, left triangular inferior frontal gyrus (IFG), right superior marginal gyrus, left anterior cingulate gyrus, left rectus, left orbital IFG, right triangular IFG, right putamen, left superior parietal gyrus and right precuneus. Besides, multimodal meta-analysis identified left middle frontal gyrus, left orbital IFG, and right putamen as the overlapped regions.

**Conclusion:**

Our results confirm that IBS patients have abnormal alterations in rs-FC and GM, and reveal brain regions with both functional and structural alterations. These results may contribute to understanding the underlying pathophysiology of IBS.

**Systematic review registration:**

https://www.crd.york.ac.uk/prospero, identifier CRD42022351342.

## 1. Introduction

Irritable bowel syndrome (IBS) is a prevalent chronic functional gastrointestinal disorder manifested by recurrent abdominal pain and alterations in stool form or frequency (Ford et al., 2020). Previous studies have revealed that the brain-gut axis plays an important role in the occurrence of IBS (Koloski et al., 2016). This axis regulates gastrointestinal motility, visceral sensitivity, stress response, and cognitive function, and is closely related to central neural mechanism (Price et al., 2006). Moreover, a recent meta-analysis (Su et al., 2022) also confirmed that IBS patients had abnormal local spontaneous functional activity in several parts of the brain. Considering the high global prevalence (approximate 10–15%) (Drossman et al., 2002) and the significant impact of IBS on health-related quality of life (Gralnek et al., 2000), it is necessary to explore the pathological mechanism of IBS. However, the neural mechanisms of IBS remain to be further elucidated by examining brain functional and structural alterations.

Resting-state functional magnetic resonance imaging (rs-fMRI) is a promising task-independent tool which has been used to analyze the underlying neural mechanism of various diseases (including IBS) from the perspective of functional and structural abnormalities (Lowe et al., 2000; Brady et al., 2016; Bi et al., 2021a,b; Zhao et al., 2021). From the perspective of function, resting-state functional connectivity (rs-FC) is one of the most frequently used methods in rs-fMRI studies (Fox and Greicius, 2010). Quantizing functional connectivity between anatomically different brain regions, FC can identify the connectivity patterns of distributed brain networks, such as default mode network (DMN). DMN is a set of widely investigated brain regions in rs-fMRI studies and reflects the baseline mode of brain function (Raichle et al., 2001). As previous studies mentioned, DMN is preferentially affected by chronic pain, which is a core symptom of IBS (Farmer et al., 2012; Paine, 2021). Therefore, DMN may be an important influencing factor in the pathogenesis of IBS. Other studies also demonstrated the essential role played by DMN in IBS (Liu et al., 2016; Qi et al., 2016). Some important nodes in DMN play key roles in brain-gut interactions in functional gastrointestinal syndrome (Kano et al., 2018). And some researchers have already realized the significance of DMN in patients with IBS by conducting studies specifically focused on DMN. For instance, Qi et al. (2016) found that IBS patients exhibit non-optimized topological characteristics of the DMN compared with healthy controls (HCs), and the average FC of DMN correlates negatively with symptom severity. While Skrobisz et al. (2020) discovered that, compared with HCs, patients with IBS and functional dyspepsia showed abnormal connectivity in DMN.

In addition to functional alterations, abnormal gray matter (GM) structural changes in IBS patients have also been reported using voxel-based morphometry (VBM) (Zhao et al., 2018; Li et al., 2020; Öhlmann et al., 2021). VBM method can detect structural differences throughout the brain and is a time-efficient, automated and unbiased voxel-based method (Yasuda et al., 2010). Some studies found that GM structural changes in IBS patients might relate to pain perception and gastrointestinal symptoms. For example, a previous study found that greater gastrointestinal symptoms correlated with reduced GM volume in the occipital pole in patients with IBS (Öhlmann et al., 2021). Meanwhile, another study found that decreased GM volume in bilateral thalamus was associated with increased pain thresholds in girls with IBS (Bhatt et al., 2019).

However, previous findings of rs-FC of DMN and VBM analyses in IBS patients were inconsistent. For example, both increased and decreased FC was found between right hippocampus and superior parietal gyrus (Li et al., 2013; Geng et al., 2021). For GM structural changes, some researchers only found decreased GM (Li et al., 2020), while others found both decreased and increased GM (Zhao et al., 2018; Öhlmann et al., 2021). These divergent results may be due to disease heterogeneity, small sample sizes, and sample selection differences (Fond et al., 2014; Li et al., 2020). Thus, it is necessary to conduct a meta-analysis to identify consistent abnormalities in IBS patients and to facilitate targeted treatments. In addition, since combining findings from different modalities can provide a more accurate differential analysis and can explore whether these functional changes indicate pure hemodynamic differences or reflect underlying morphological differences (Mayer et al., 2015), we integrate the multimodal findings to detect both functional and structural alterations in IBS patients.

Though a recent meta-analysis by Su et al. (2022) regarding regional spontaneous functional activity in IBS patients has been conducted, it focused exclusively on local brain activities instead of whole-brain FC. Moreover, this meta-analysis did not take GM changes into consideration. Therefore, the current study aims to seek consistent alterations in DMN connectivity as well as GM anatomic changes in IBS through whole-brain rs-FC and VBM. The findings of this meta-analysis might consolidate prior divergent results and facilitate the identification of potential IBS biomarkers in future studies.

## 2. Methods

### 2.1. Literature search, selection criteria, and quality assessment

This meta-analysis has been registered in the PROSPERO International Prospective Register of Systematic Reviews^1^ with the registration number “CRD42022351342” (available at https://www.crd.york.ac.uk/prospero/display_record.php?RecordID=351342).

To conduct FC and VBM meta-analyses, a systematic literature search was performed according to the standard preferred reporting items for systematic reviews and meta-analyses (PRISMA) procedure (Moher et al., 2009; Wu et al., 2020; Yan et al., 2023). We searched in PubMed, Embase, Web of Science, Chinese National Knowledge Infrastructure (CNKI), Wanfang Database (WF) and Scoups for published research up to August 12, 2023. For FC meta-analysis, the following keywords and their combinations were used: “resting state functional connectivity” or “resting state FC” or “rs-FC” or “ICA” or “independent component analysis” and “IBS” or “irritable bowel syndrome” and “DMN” or “default mode network.” For VBM analysis, the keywords included: “voxel-based morphometry,” “VBM,” “IBS,” and “irritable bowel syndrome.” In addition, the references lists of the retrieved studies were checked for potentially eligible studies.

The selection criteria were as follows: (1) Patients were diagnosed with irritable bowel syndrome based on any diagnostic criteria (Zhang et al., 2018); (2) Analysis was performed using seed-based FC or independent component analysis or VBM in the whole brain level; (3) A direct brain imaging comparison was conducted between IBS patients and healthy controls (HCs); (4) Peak coordinates (Talairach or Montreal Neurological Institute (MNI)) were reported, if the results showed significant statistical differences; and (5) The study was published in a peer-reviewed English or Chinese language journal.

Studies were excluded if they met one of the following conditions: (1) Studies were review articles, conference abstracts, or non-human research; (2) IBS patients diagnosed with comorbid neurological or psychiatric conditions; (3) The baseline comparison between IBS patients and HCs was not provided; (4) The analysis was not performed at the whole-brain level; and (5) If different studies had overlapping samples, only the study with the largest sample was included.

In addition, the quality of each study included in this meta-analysis was assessed using an 11-point checklist based on the previous meta-analyses, which include the assessment of demographic and clinical characteristics, scanner parameters, and analysis details (Cheng et al., 2020). According to these criteria, two researchers independently conducted the literature searches, study selection, data extraction, and quality evaluation. Extracted data include demographic and clinical characteristics of participants (sample size, age, gender, and illness duration), scanner parameters and basic methodological information (methods and software using for fMRI analysis, and statistical threshold information) (Table 1). We also extracted the coordinates of significant findings and statistical values related to effect size for SDM calculations. Any inconsistent opinions were discussed and settled through consultation. If no agreement could be reached, it was decided by a third author.

**TABLE 1:** Studies included in the rs-FC meta-analysis.

References	Subjects (females)		Mean age (SD)		Methods	Seed region	Illness duration (SD)^1^	Scanner	FWHM	Software	Threshold	Quality scores (out of 11)
	Patients	Controls	Patients	Controls								
Hubbard et al., 2016	17 (13)	17 (13)	16.44 (1.73)	16.29 (1.83)	FC	PCC^5^	43.44 (30.24)	3.0T	8 mm	SPM8	*p* < 0.05, corrected	11
Li et al., 2013	21 (7)	21 (10)	41.8 (11.9)	35.9 (14.8)	FC	Hippo	59.04 (36.84)	1.5T	4 mm	DPARSF	*p* < 0.05, corrected	8.5
Longarzo et al., 2017	19 (13)	26 (16)	39.6 (13.5)	40.1 (15.3)	FC	PCC^6^	NA	3.0T	NA	SPM8	*p* < 0.05, corrected	10
Chen et al., 2021	38 (20)	36 (26)	34.36 (9.53)	31.67 (8.85)	FC	Precuneus^7^	229.32 (83.76)	3.0T	6 mm	DPABI	*p* < 0.05, corrected	11
Icenhour et al., 2017	41 (41)	20 (20)	^2^	32.25 (2.2)	ICA	NA	NA	1.5T	NA	GIFT, SPM8	*p* < 0.05, corrected	10.5
Gupta et al., 2014	58 (28)	110 (72)	^3^	^4^	ICA	NA	NA	3.0T	5 mm	GIFT,SPM8	*p* < 0.005, corrected	10.5

FC, functional connectivity; ICA, independent component analysis; SD, standard deviation; NA, not available; FWHM, full width at half maximum; SPM, statistical parametric mapping; DPARSF, data processing assistant for resting-state fMRI; DPABI, data processing and analysis for brain imaging; GIFT, group independent component analysis of fMRI toolbox; PCC, posterior cingulate cortex; Hippo, hippocampus.

^1^The unit of illness duration is in months.

^2^The mean age of normosensitive IBS patients is 33.25 (2.27), and hypersensitive IBS patients is 36.48 (2.71).

^3^The mean age of male IBS patients is 37.28 (10.75), and female IBS patients is 30.65 (10.71).

^4^The mean age of male controls is 35.95 (12.97), and female controls is 29.39 (9.93).

^5^The MNI coordinate of the PCC seed in this study is (15, −29, 38).

^6^The MNI coordinate of the PCC seed in this study is (−5, −49, 40).

^7^The MNI coordinate of Precuneus seed in this study is (−3, −54, 18).

### 2.2. Voxel-wise meta-analysis

Functional connectivity and VBM meta-analyses were processed using the anisotropic effect-size signed differential mapping (AES-SDM) version 5.15^2^ (Radua et al., 2014). First, the peak coordinates (both positive and negative) and corresponding effect sizes of statistically significant clusters were extracted from each included study. Then, an effect-size map of the individual study was created using an anisotropic unnormalized Gaussian kernel (Radua et al., 2014). Finally, the mean of the study maps was calculated with a random-effects model, and weighted by the sample size, the intra-study variance, and inter-study heterogeneity (Radua et al., 2012). We applied the recommended AES-SDM kernel size and thresholds, namely, 20 mm full width at half maximum (FWHM), voxel *p* < 0.005, peak height SDM-*Z* > 1, and cluster extent > 10 voxels (Radua et al., 2014). Images were displayed on a standardized anatomical template in the MNI space. Considering the influence of other important brain networks, we also conducted two additional meta-analyses for salience network and sensorimotor network (see Supplementary material).

### 2.3. Analyses of sensitivity, heterogeneity, and publication bias

A jackknife sensitivity analysis was performed to test the replicability of the results (Radua and Mataix-Cols, 2009). The identical analysis was repeated by discarding one of the included studies each time to assess if a result was robust and reliable. Besides, using a random-effect model with Q statistics, a heterogeneity analysis was performed to detect unexplained inter-study variability. And the threshold of this heterogeneity analysis was set at voxel *p* < 0.005, peak height *Z* > 1, and cluster extent > 10 voxels (Radua et al., 2014). Additionally, we implemented an Egger test to explore the probability of publication bias for each cluster through the values of the peaks in the main meta-analysis. Publication bias indicates the tendency to report statistically significant results more frequently than non-significant findings (Sterne et al., 2011). The results showing *p* < 0.05 are considered to be statistically significant (Egger et al., 1997).

### 2.4. Multimodal analysis

To identify the overlapping brain regions that are significant in both functional and structural modalities, a multimodal approach was conducted by AES-SDM. The probability maps of VBM and functional response alterations would be combined to cross-validate the union alteration in both modalities (Nichols et al., 2005; Radua et al., 2013). The voxel-level threshold was set at *p* < 0.0025 as recommended (Radua et al., 2013).

### 2.5. Meta-regression analysis

Considering the influence of gender, meta-regression was conducted to examine the impact of gender on functional and structural alterations. The significant level was set at *p* < 0.0005, *Z* > 1, and cluster size > 10 voxels to reduce spurious findings (Radua and Mataix-Cols, 2009; Zhou et al., 2017).

## 3. Results

### 3.1. Included studies, sample, and analytic characteristics

**FIGURE 1:**
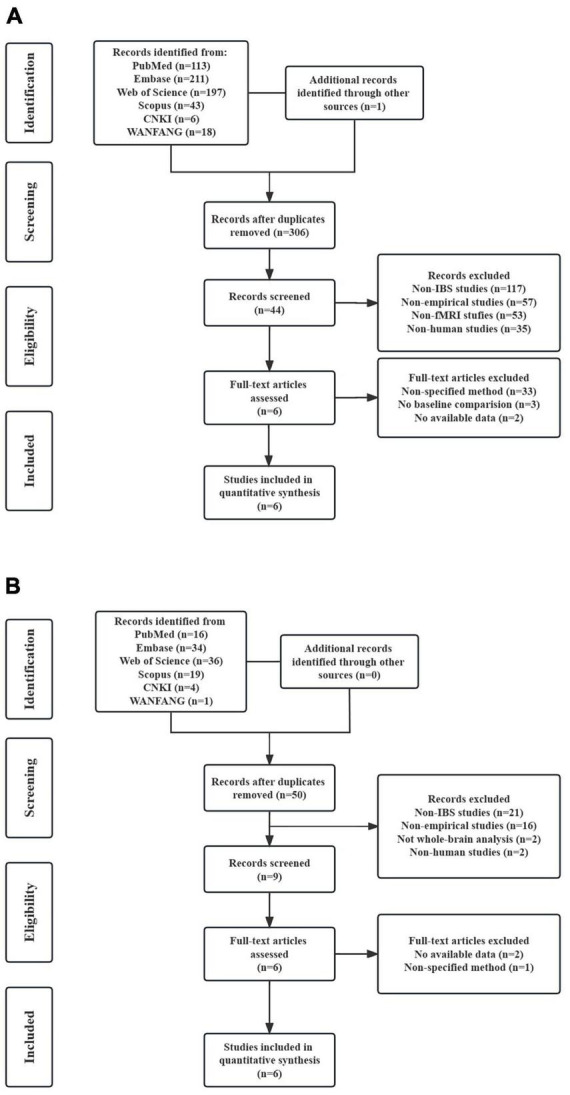
Preferred reporting items for systematic reviews and meta-analyses (PRISMA) flow diagram for literature identification and exclusion. **(A)** Flow diagram of study identification and selection for resting-state functional connectivity; **(B)** flow diagram of study identification and selection for voxel-based morphometry.

In the FC meta-analysis, 6 studies were finally included, consisting of 194 patients with IBS (122 females and 72 males; mean age = 34.139) and 230 healthy controls (157 females and 73 males; mean age = 31.916). All studies had well-matched control groups. The mean quality score of FC studies was 10.25, and the mean score of VBM studies was 10.25. Quality assessment items that deducted the most scores of these studies were lack of desired information (including medication status, illness duration, comorbidity and severity of illness) and limitation discussion. Notably, the study of Gupta et al. (2014) and Icenhour et al. (2017) used ICA analysis, and the other four studies all applied a seed-based FC analysis. The DMN seeds used in FC analysis were shown in Table 1. As for the VBM meta-analysis, 6 studies were selected, with 189 IBS patients (150 females and 39 males) and 162 healthy controls (127 females and 35 males). The demographic and clinical characteristics, and analytic methods of included VBM studies are shown in Table 2. The process for literature identification and screening is presented in Figure 1.

**TABLE 2:** Studies included in the VBM meta-analysis.

References	Subjects (females)		Mean age (SD)		Illness duration (SD)^1^	Scanner	FWHM	Software	Threshold	Quality scores (out of 11)
	Patients	Controls	Patients	Controls						
Seminowicz et al., 2010	55 (55)	48 (48)	32.2 (12.3)	31.2 (12.3)	133.2 (92.76)	3.0T	NA	SurfStat	*p* < 0.1, corrected	10
Fang, 2013	19 (13)	20 (14)	38.39 (7.65)	36.48 (8.23)	NA	1.5T	12 mm	SPM5	*p* < 0.05, uncorrected	10
Öhlmann et al., 2021	23 (23)	23 (23)	46.91 (10.92)	43.74 (12.17)	NA	3.0T	8 mm	SPM12	*p* < 0.05, corrected	10.5
Li et al., 2020	49 (20)	36 (10)	^2^	31.67 (8.85)	^3^	3.0T	8 mm	DPABI	*p* < 0.05, corrected	10.5
Bhatt et al., 2019	32 (32)	26 (26)	14.54 (2.91)	12.05 (3.13)	NA	3.0T	5 mm	FSL	*p* < 0.05, corrected	10.5
Davis et al., 2008	11 (7)	9 (6)	24–50	30–58	NA	1.5T	10 mm	SPM2	*p* < 0.001, uncorrected	10

SD, standard deviation; NA, not available; FWHM, full width at half maximum; SPM, statistical parametric mapping; DPABI, data processing and analysis for brain imaging; FSL, FMRIB’s Software Library.

^1^The unit of illness duration is in months.

^2^The mean age of patients with IBS who lacked depressive symptoms is 32.29 (9.96), and for patients with IBS who had depressive symptoms is 36.36 (7.31).

^3^The illness duration of patients with IBS who lacked depressive symptoms is 18.57 (4.00) months, and for patients with IBS who had depressive symptoms is 20.64 (9.01) months.

### 3.2. Main meta-analysis

The meta-analysis revealed abnormal rs-FC between DMN and other brain regions. As presented in Table 3, patients with IBS showed increased rs-FC in left inferior parietal gyrus (IPG), left lingual gyrus, right angular gyrus, and right precuneus. Meanwhile, decreased rs-FC was detected in the right amygdala, right median cingulate gyrus (MCG), and left hippocampus.

**TABLE 3:** Clusters showing rs-FC and GM differences in patients with IBS compared with HCs.

Location	BA	Local peak (MNI)			*P*-value	SDM-*Z*-value	Voxels	Sensitivity analysis	Egger test (*P*-value)	Heterogeneity (k)
		x	y	z						
**FC meta-analysis**										
IBS > HC										
Parietal_Inf_L	40	−36	−56	50	4.3E-05	2.143	845	5 out 6	0.207	Sig. (55)
Lingual_L	NA	−2	−56	6	3.4E-05	2.182	497	5 out 6	0.099	Sig. (38)
Angular_R	39	48	−56	28	0.00047	1.742	189	5 out 6	0.007*	ns
Precuneus_R	23	10	−56	28	0.00035	1.775	91	5 out 6	0.007*	ns
IBS < HC										
Amygdala_R	48	34	0	−18	1.8E-05	−1.428	789	5 out 6	0.264	ns
Cingulum_Mid_R	32	6	18	40	0.00037	−1.011	594	5 out 6	0.365	ns
Hippocampus_L	NA	−24	−32	10	0.00027	−1.056	81	5 out 6	0.202	ns
**VBM meta-analysis**										
IBS > HC										
Fusiform_L	37	−34	−64	−10	0.00021	1.835	389	5 out 6	0.54	ns
Fusiform_R	37	24	−40	−18	0.00132	1.436	248	5 out 6	0.18	ns
Fusiform_L	37	−28	−34	−16	0.00137	1.435	248	5 out 6	0.18	ns
Frontal_Inf_Tri_L	48	−42	18	30	0.00044	1.691	115	5 out 6	0.155	ns
SupraMarginal_R	48	46	−26	26	0.00218	1.39	14	5 out 6	0.181	ns
**IBS < HC**										
Cingulum_Ant_L	10	0	50	4	1.6E-05	−2.307	841	6 out 6	0.767	ns
Rectus_L	11	−14	22	−12	1E-05	−2.434	358	6 out 6	0.111	ns
Frontal_Inf_Orb_L	47	−36	38	−12	0.00019	−2.014	103	6 out 6	0.141	ns
Frontal_Inf_Tri_R	45	52	36	24	0.0008	−1.851	64	4 out 6	0.169	ns
Putamen_R	48	18	8	−8	0.00247	−1.642	35	5 out 6	0.164	ns
Parietal_Sup_L	2	−32	−48	64	0.00328	−1.588	11	3 out 6	0.225	ns
Precuneus_R	7	8	−72	58	0.00174	−1.713	10	4 out 6	0.148	ns

*The publication bias was detected in the clusters of right angular gyrus and right precuneus. MNI, Montreal Neurological Institute; BA, Brodmann area; NA, not available; ns, non-significant; Sig., significant; k, voxel extent of the cluster of heterogeneity; Parietal_Inf_L, left inferior parietal gyrus; Lingual_L, left lingual gyrus; Angular_R, right angular gyrus; Precuneus_R, right precuneus; Amygdala_R, right amygdala; Cingulum_Mid_R, right median cingulate gyrus; Hippocampus_L, left hippocampus; Fusiform_L, left fusiform gyrus; Fusiform_R, right fusiform gyrus; Frontal_Inf_Tri_L, left triangular inferior frontal gyrus; SupraMarginal_R, right superior marginal gyrus; Cingulum_Ant_L, left anterior cingulate gyrus; Rectus_L, left gyrus rectus; Frontal_Inf_Tri_R, right triangular inferior frontal gyrus; Putamen_R, right putamen; Parietal_Sup_L, left superior parietal gyrus; Precuneus_R, right precuneus.

Gray matter structural abnormalities were also observed in the meta-analysis. Bilateral fusiform gyrus, left triangular IFG and right superior marginal gyrus (SMG) showed increased GM in patients with IBS compared with HCs. However, the left anterior cingulate gyrus (ACG), left gyrus rectus, left orbital inferior frontal gyrus, right triangular IFG, right putamen, left superior parietal gyrus (SPG) and right precuneus showed decreased GM in patients with IBS.

### 3.3. Analyses of sensitivity, heterogeneity, and publication bias

For abnormal regions in FC meta-analysis, jackknife sensitivity analysis revealed that all seven clusters remained significant in five combinations out of six datasets, as shown in Table 3. And heterogeneity analysis showed that left IPG and left lingual gyrus had significant heterogeneity. As for the Egger test, right angular gyrus and right precuneus showed the presence of publication bias.

Meanwhile, the major results of the VBM meta-analysis remain reliable after sensitivity analysis. Specifically, all clusters showing increased GM (including bilateral fusiform, left triangular IFG and right SMG) in patients with IBS were significant in all but one combination. Decreased GM in left ACG, left gyrus rectus and left orbital IFG were detected in all combinations, while right putamen was detected in five combinations, followed by right precuneus and right triangular IFG detected in four combinations. And left SPG was only significant in three combinations. All significant brain regions in VBM meta-analysis did not present significant publication bias and the presence of heterogeneity.

### 3.4. The conjunction of dysconnectivity and abnormal GM in IBS

**FIGURE 2:**
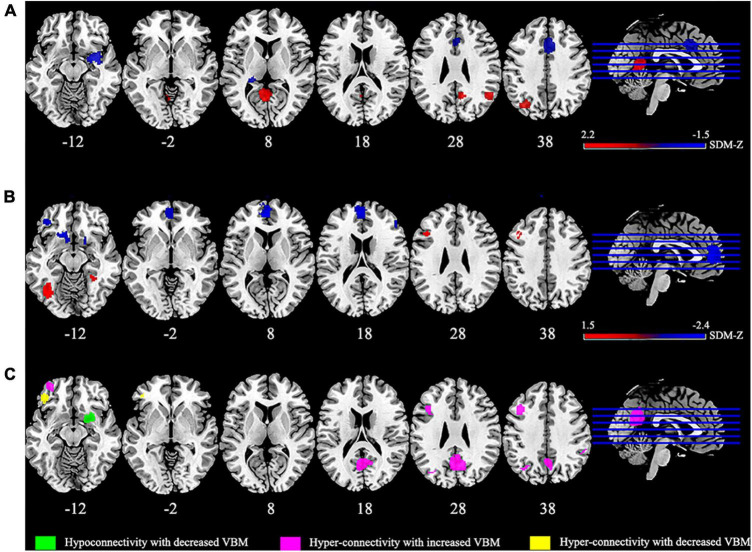
The results of meta-analysis. **(A)** Clusters showing functional connectivity (FC) differences in patients with irritable bowel syndrome (IBS) compared with healthy controls (HCs) (red denotes increased FC, blue denotes decreased FC). **(B)** Clusters showing gray matter (GM) differences in IBS patients compared with HCs (red denotes increased GM, blue denotes decreased GM). **(C)** The overlap regions showed significant functional and gray matter structural changes between patients with IBS and HCs.

As presented in Figure 2, multimodal meta-analysis showed that hyper-connectivity converged with increased GM was significant in left middle frontal gyrus (MFG), and hyper-connectivity converged with decreased GM in left orbital IFG. Besides, right putamen showed hypoconnectivity combined with GM decrease.

### 3.5. Meta-regression analysis

No significant associations were found between gender and functional as well as structural changes.

## 4. Discussion

This meta-analysis synthesizes diverse results from rs-FC of DMN and VBM results in IBS patients. The voxel-based meta-analysis showed rs-FC changes mainly in DMN and limbic regions. In addition, abnormal structural brain changes were detected in regions mainly participating in pain and emotion processing (including the fusiform gyrus, IFG, SMG, ACG, rectus, SPG, putamen and precuneus). The multimodal analysis revealed that GM changes were closely integrated with functional alterations in regions responsible for attention, emotion and pain processing (i.e., MFG, orbital IFG and right putamen).

For FC meta-analysis, our results found that increased FC was mainly within DMN regions, including left IPG, right angular gyrus and right precuneus. Several studies have also found abnormal brain activities of DMN in patients with IBS (Qi et al., 2016; Bhatt et al., 2022), and Letzen et al. (2013) found that DMN plasticity was sensitive to analgesic effects which further confirms its important role in pain. Previous study demonstrated that DMN activity increased when attention is engaged in mind wandering and decreased when attention is captured by pain (Kucyi and Davis, 2015). Additionally, DMN is also involved in pain-related attention and information gathering process (Cavanna and Trimble, 2006; Ploner et al., 2011). Therefore, we speculated that increased FC within DMN could imply deficits in pain-related visceral attention and sensations. Besides, our study also found increased FC in visual area (left lingual gyrus), consistent with a previous study that also demonstrated abnormal brain activities in visual cortex in patients with IBS (Lee et al., 2012). Meanwhile, patients with chronic pain showed altered functional activity in visual cortical areas as well (Li et al., 2018; Messina et al., 2022). This could be associated with abnormality in pain perception in patients with IBS. To be noted that right angular gyrus and right precuneus showed significant publication bias, while left IPG and left lingual showed significant heterogeneity, which could be due to clinical differences or demographic information and should be interpreted with caution (Sterne et al., 2011).

In the present study, decreased connectivity was found between DMN and limbic system (including amygdala, hippocampus and MCG). The limbic system links visceral states and emotions to cognition and behavior, and its dysfunction is associated with emotion dysregulation, memory impairment and cognitive deficits (Catani et al., 2013). Previous studies have discovered dysfunction of the hippocampal-amygdala circuit and abnormal hippocampus activities during heterotopic stimulation and cognitive task in IBS patients compared with HCs (Wilder-Smith et al., 2004; Aizawa et al., 2012). Besides, previous studies found that MCG took part in the processing of emotional and cognitive responses to painful information, and the local brain activities in the MCG were related to the disease duration (Frot et al., 2008; Ma et al., 2015). Taken together, visceral pain was detected and adjusted inappropriately through emotional arousal and homeostatic afferent networks (including the amygdala and MCG regions), then the conditioned fear of pain will be formatted by the hippocampus, making this process a vicious circle (Tillisch et al., 2011; Murray et al., 2021).

As for VBM meta-analysis, changed GM was mainly detected in regions involved in pain processing, including pain perception, transmission and interpretation. The altered brain regions included a wide range of areas in the frontal, parietal and temporal lobes. Namely, SMG, SPG and precuneus belong to the parietal lobe, while fusiform belong to the temporal lobe, which is involved in sensory information perception, imagination and integration (Hyvärinen, 1982; Spagna et al., 2021). Besides, the gyrus rectus connects the sensory integration network and visceral-motor centers (Öngür and Price, 2000). Accordingly, the abnormal changes in these regions will not only influence the reception of pain sensory inputs but also disturb the visceromotor outputs. Previous studies have found reduced volumes and abnormal activities in these regions in IBS patients (Labus et al., 2014; Guleria et al., 2017), and pain sensitivity questionnaire scores were found to have a positive correlation with gray matter volume in fusiform gyrus (Ruscheweyh et al., 2018).

After experiencing initial pain, this feeling could still be modified by top-down cognitive controls, which link to regions involved in high-level cognition and affects (Garcia-Larrea and Bastuji, 2018). And this also reflected in our results. ACG and IFG play important roles in cognitive control and negative affects, which are all influence factors for IBS development (Beer et al., 2006; Shackman et al., 2011; Obermann et al., 2013). A prior study pointed out that the reduction of gray matter volume in frontal gyrus was associated with impaired cognitive function (Luo et al., 2015). Previous studies investigating pain disorders found reduced GM in ACG as well (Obermann et al., 2013; Kregel et al., 2015). Putamen is another region that involved in modulation and emotional responses to pain (Scott et al., 2006; Borsook et al., 2010). Azqueta-Gavaldon et al. (2020) found that patients with complex regional pain syndrome had decreased gray matter density in the putamen and that increased connectivity between putamen and pre-/post-central gyrus was associated with more severe clinical pain. The interaction among these brain regions may provide a possible explanation for the abnormal visceral perception and visceral hypersensitivity in IBS.

Through multimodal conjunction analysis, the consistent regions which showed dysconnectivity converged with changed GM were detected, including hyper-connectivity combined with increased GM in left MFG, hyper-connectivity combined with decreased GM in left orbital IFG, and hypoconnectivity combined with decreased GM in the right putamen. Previous study pointed out that brain functional and microstructural changes in IBS patients were particularly located in regions associated with the processing, integration and modulation of sensory information (Mayer et al., 2015), which are consistent with our results. As illustrated above, IFG and putamen are related to pain integration and modulation, while MFG is a part of prefrontal cortex and implicated in attention modulation (Japee et al., 2015), and a previous meta-analysis of patients with IBS also found abnormal brain activities in this area (Su et al., 2022). Researchers also elucidated that its abnormal activity might be associated with selective attention to stomach sensations in patients with functional dyspepsia (Zeng et al., 2011). Our results showed functional as well as structural changes in MFG, and this could imply that the gastrointestinal symptoms in IBS patients may relate to inappropriate attention toward stomach sensation. On the whole, these results further indicated that IBS is a psychosomatic disorder, and psychological factors may be important moderators in patients with IBS (Fond et al., 2014).

Some limitations should be taken into account when interpreting the results in our study. First, the present meta-analysis only contains six studies of FC and six studies of VBM, which is a relatively small sample size. Considering the difficulty of recruiting eligible patients and the diversity of research methods in rs-fMRI studies, it is challenging to incorporate a large number of methods-specific articles and this seems to be a common dilemma in meta-analysis of fMRI studies. Nevertheless, our study could be seen as an exploratory study to provide the first meta-analytic evidence of DMN together with VBM alterations in IBS. Future studies should include more up-to-date studies and confirm these findings. Second, our VBM meta-analysis only includes studies focusing on gray matter changes, therefore, future studies should conduct meta-analysis of diffusion tensor imaging in IBS patients to explore white matter changes. Furthermore, our study only focused on DMN, future meta-analyses should take other important brain networks into consideration as well.

## 5. Conclusion

Our study presents evidence on dysconnectivity of DMN and aberrant changes of GM in IBS. IBS patients exhibited disrupted associations within DMN and between DMN and the limbic system. Besides, altered GMV was demonstrated in regions mostly associated with pain and emotion processing. And multimodal analysis found that hyper/hypo-connectivity converged with GM changes was located in brain regions involved in attention, emotion and pain processing. These results could help us to further understand the pathophysiology of IBS and motivate future studies to identify potential IBS biomarkers.

## Data availability statement

The original contributions presented in the study are included in the article/Supplementary material, further inquiries can be directed to the corresponding authors.

## Author contributions

JR, YY, and XJ contributed to the conception of the study. MZ, SH, and JW conducted screening, data extraction, and statistical analysis. ML and YG contributed to analysis and manuscript preparation. MZ and ZH wrote the first draft of the manuscript. ZH and CA helped review and editing the manuscript. All authors read and approve the final manuscript.

## Footnotes

1
https://www.crd.york.ac.uk/prospero

2
http://www.sdmproject.com
