# Supplementary material for: Functional changes of default mode network and structural alterations of gray matter in patients with irritable bowel syndrome: a meta-analysis of whole-brain studies
Source: Front Neurosci. 2023 Oct 24;17:1236069. doi: 10.3389/fnins.2023.1236069 (PMC10627928; doi:10.3389/fnins.2023.1236069)
Supplement: Supplementary file 1 [file Data_Sheet_1.docx]

Supplementary Material

**Supplementary Tables and Figures**

Table S1: Studies included in the rs-FC sensorimotor network meta-analysis

| Study | Subjects (females) | | Mean age (SD) | | Methods | Seed region | Illness duration (SD)^1^ | Scanner | FWHM | Software | Threshold | Quality scores  (out of 11) |
| --- | --- | --- | --- | --- | --- | --- | --- | --- | --- | --- | --- | --- |
|  | Patients | Controls | Patients | Controls |  |  |  |  |  |  |  |  |
| Hubbard et al, (2016) | 17 (13) | 17 (13) | 16.44  (1.73) | 16.29  (1.83) | FC | SI | 43.44  (30.24) | 3.0T | 8mm | SPM8 | *p* < 0.05,  corrected | 11 |
| Longarzo et al, (2017) | 19 (13) | 26 (16) | 39.6  (13.5) | 40.1  (15.3) | FC | pINS | NA | 3.0T | NA | SPM8 | *p* < 0.05,  corrected | 10 |
| Chen et al, (2021) | 38 (20) | 36 (26) | 34.36  (9.53) | 31.67  (8.85) | FC | PoCG | 229.32  (83.76) | 3.0T | 6mm | DPABI | *p* < 0.05,  corrected | 11 |
| Icenhour et al, (2017) | 41 (41) | 20 (20) | ^2^ | 32.25  (2.2) | ICA | NA | NA | 1.5T | NA | GIFT, SPM8 | *p* < 0.05,  corrected | 10.5 |
| Bhatt et al, (2017) | 32 (32) | 26 (26) | 11.40  (3.01) | 10.72  (2.79) | FC | Caudate | NA | 3.0T | NA | SPM8 | *p* < 0.05,  corrected | 10 |

Abbreviations: FC, functional connectivity; ICA, independent component analysis; SD, standard deviation; NA, not available; FWHM, full width at half maximum; SPM, statistical parametric mapping; DPABI, data processing & analysis for brain imaging; GIFT, group independent component analysis of fMRI toolbox; SI, primary somatosensory cortex; pINS, posterior insula; PoCG, postcentral gyrus.

^1^ The unit of illness duration is in months.

^2^ The mean age of normosensitive IBS patients is 33.25 (2.27), and hypersensitive IBS patients is 36.48 (2.71).

Table S2: Studies included in the rs-FC sensorimotor network meta-analysis

| Study | Subjects (females) | | Mean age (SD) | | Methods | Seed region | Illness duration (SD)^1^ | Scanner | FWHM | Software | Threshold | Quality scores  (out of 11) |
| --- | --- | --- | --- | --- | --- | --- | --- | --- | --- | --- | --- | --- |
|  | Patients | Controls | Patients | Controls |  |  |  |  |  |  |  |  |
| Hubbard et al, (2016) | 17 (13) | 17 (13) | 16.44  (1.73) | 16.29  (1.83) | FC | aINS | 43.44  (30.24) | 3.0T | 8mm | SPM8 | *p* < 0.05,  corrected | 11 |
| Longarzo et al, (2017) | 19 (13) | 26 (16) | 39.6  (13.5) | 40.1  (15.3) | FC | aINS | NA | 3.0T | NA | SPM8 | *p* < 0.05,  corrected | 10 |
| Icenhour et al, (2017) | 41 (41) | 20 (20) | ^2^ | 32.25  (2.2) | ICA | NA | NA | 1.5T | NA | GIFT, SPM8 | *p* < 0.05,  corrected | 10.5 |
| Gupta et al,(2014) | 58(28) | 110(72) | ^3^ | ^4^ | ICA | NA | NA | 3.0T | 5mm | GIFT,SPM8 | p<0.005,  corrected | 10.5 |

Abbreviations: FC, functional connectivity; ICA, independent component analysis; SD, standard deviation; NA, not available; FWHM, full width at half maximum; SPM, statistical parametric mapping; DPARSF, data processing assistant for resting-state fMRI; DPABI, data processing & analysis for brain imaging; GIFT, group independent component analysis of fMRI toolbox; aINS, anterior insula.

^1^ The unit of illness duration is in months.

^2^ The mean age of normosensitive IBS patients is 33.25 (2.27), and hypersensitive IBS patients is 36.48 (2.71).

^3^ The mean age of male IBS patients is 37.28 (10.75), and female IBS patients is 30.65 (10.71).

^4^ The mean age of male controls is 35.95 (12.97), and female controls is 29.39 (9.93).

Table S3: Clusters showing rsFC differences in sensorimotor network in IBS compared with HCs.

| Location | BA | Local peak (MNI) | | | *p* value | SDM-Z value | Voxels |
| --- | --- | --- | --- | --- | --- | --- | --- |
|  |  | x | y | z |  |  |  |
| IBS>HC |  |  |  |  |  |  |  |
| Rolandic_Oper_L | 48 | -36 | -22 | 2 | 0.001078069 | 1.379 | 205 |
| Rolandic_Oper_R | 48 | 50 | -24 | 18 | 0.001078069 | 1.380 | 256 |
| Supp_Motor_Area_L | NA | -2 | -18 | 50 | 0.000005960 | 1.938 | 844 |
| Precentral_L | 6 | -50 | -2 | 44 | 0.001078069 | 1.379 | 49 |
| Precentral_R | 6 | 24 | -16 | 66 | 0.001078069 | 1.379 | 43 |
| IBS<HC (none) |  |  |  |  |  |  |  |

Abbreviation: MNI, Montreal Neurological Institute; Rolandic_Oper_L, left rolandic operculum; Rolandic_Oper_R, right rolandic operculum; Supp_Motor_Area_L, left supplementary motor area; Precentral_L, left precentral gyrus; Precentral_R, right precentral gyrus.

Table S4: Clusters showing rsFC differences in salience network in IBS compared with HCs.

| Location | BA | Local peak (MNI) | | | *p* value | SDM-Z  value | Voxels |
| --- | --- | --- | --- | --- | --- | --- | --- |
|  |  | x | y | z |  |  |  |
| IBS>HC |  |  |  |  |  |  |  |
| SupraMarginal_R | 40 | 58 | -38 | 38 | 0.000154555 | 1.833 | 314 |
| Cingulum_Mid_R | 24 | 6 | 26 | 30 | 0.000154555 | 1.833 | 164 |
| Frontal_Med_Orb_R | 10 | 4 | 42 | -4 | 0.000123620 | 1.833 | 161 |
| Insula_L | 48 | -34 | 14 | -6 | 0.000154555 | 1.832 | 128 |
| Frontal_Inf_Orb_R | 47 | 34 | 22 | -12 | 0.000154555 | 1.832 | 97 |
| Cingulum_Ant_L | 32 | -8 | 40 | 18 | 0.000318706 | 1.822 | 48 |
| Putamen_L | NA | -22 | 6 | 2 | 0.000548601 | 1.807 | 24 |
| IBS<HC (none) |  |  |  |  |  |  |  |

Abbreviation: MNI, Montreal Neurological Institute; SupraMarginal_R, right supramarginal gyrus; Cingulum_Mid_R, right median cingulate gyrus; Frontal_Med_Orb_R, right orbital medial frontal gyrus; Insula_L, left insula; Frontal_Inf_Orb_R, right orbital inferior frontal gyrus; Cingulum_Ant_L, left anterior cingulate gyrus; Putamen_L, left putamen.


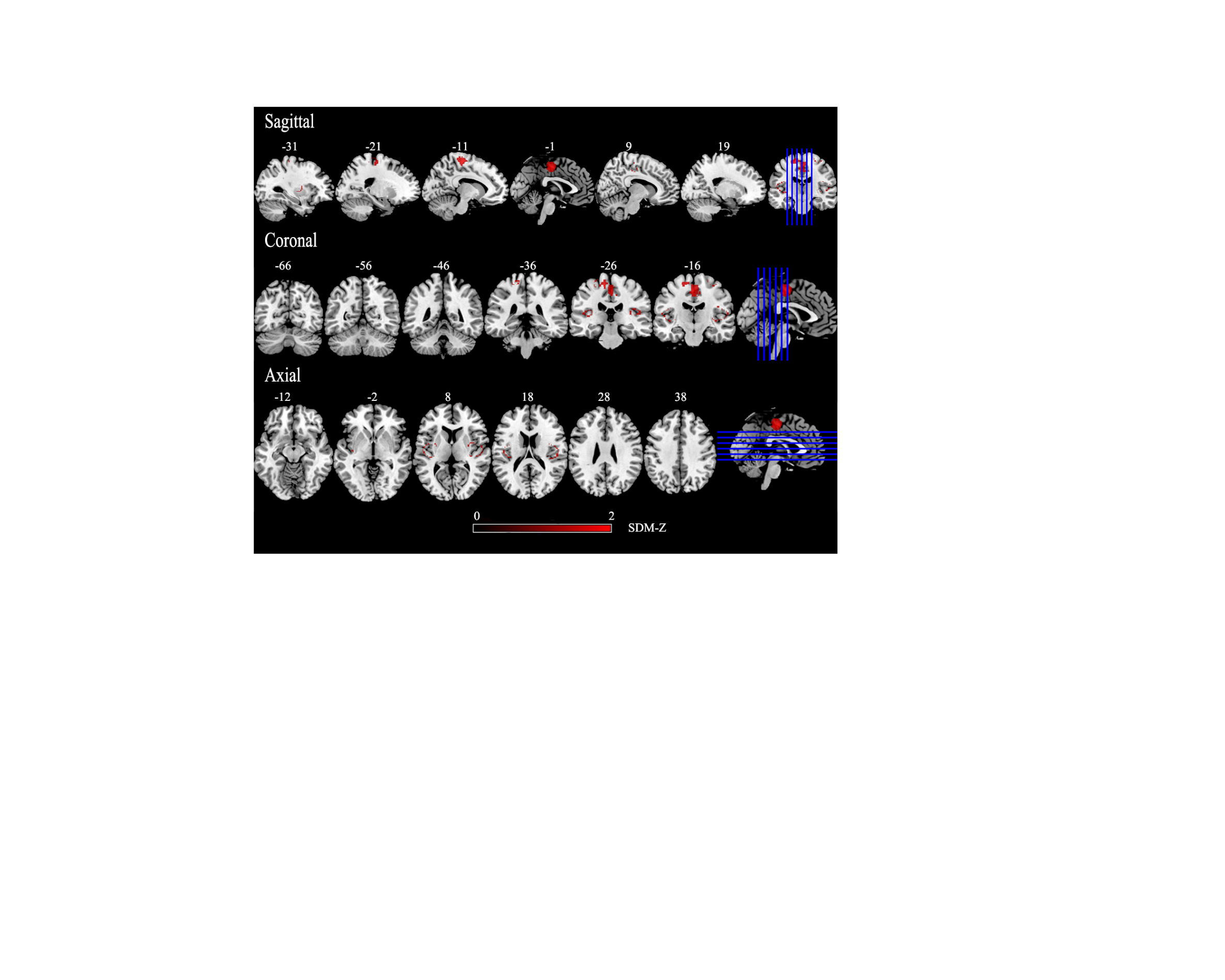


Figure S1: Clusters showing rsFC differences in sensorimotor network in IBS compared with HCs. The areas of increased (red) FC in meta-analysis. The color bar indicates the maximum and minimum SDM-Z value.


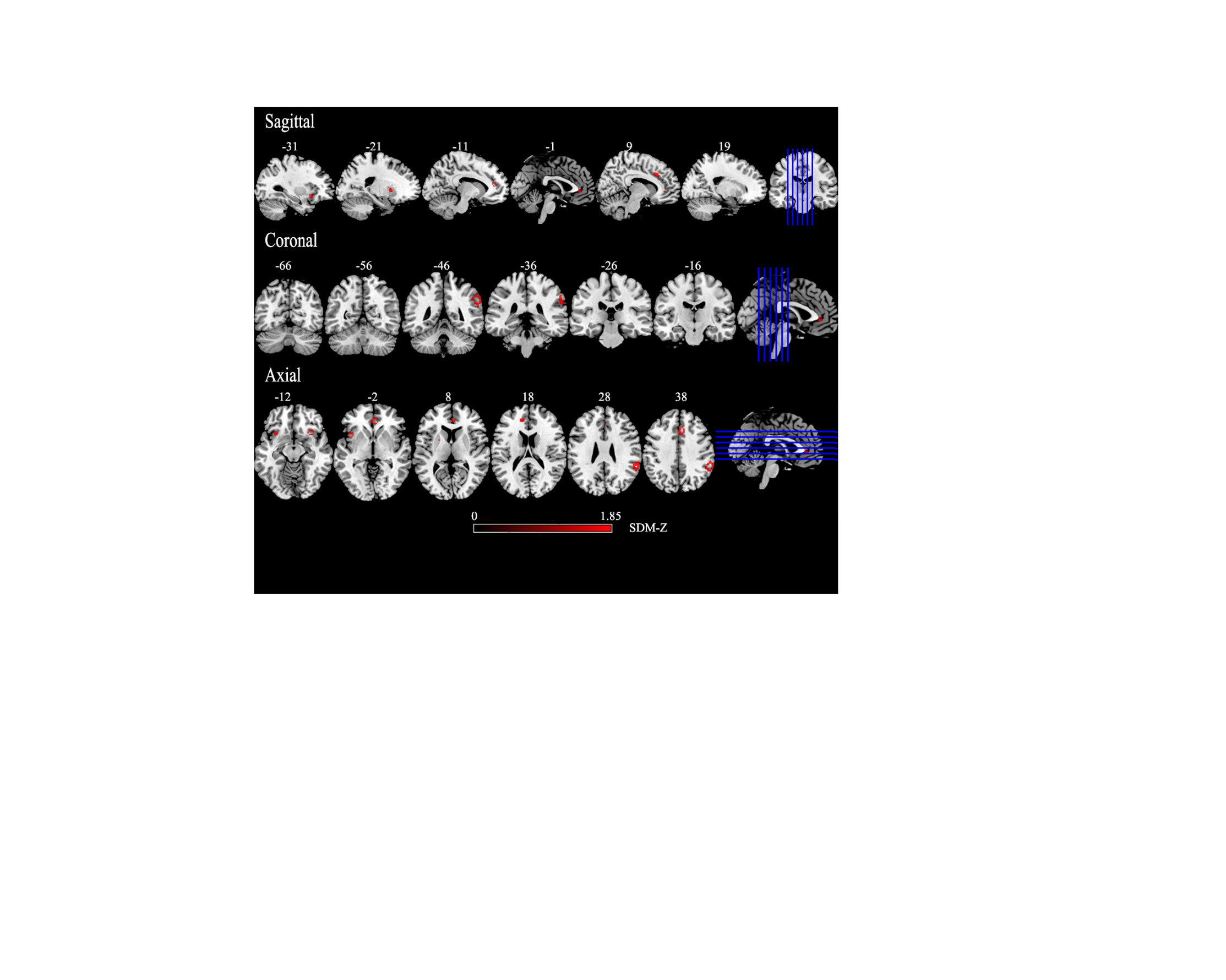


Figure S2: Clusters showing rsFC differences in salience network in IBS compared with HCs. The areas of increased (red) FC in meta-analysis. The color bar indicates the maximum and minimum SDM-Z value.

**Reference**

Bhatt, R., Gupta, A., Labus, J.S., Zeltzer, L.K., Tsao, J., and Tillisch, K. (2017). Altered brain structure and functional connectivity and its relation to pain perception in female adolescents with irritable bowel syndrome. *Gastroenterology* 152(5)**,** S727.

Chen, X.F., Guo, Y., Lu, X.Q., Qi, L., Xu, K.H., Chen, Y., et al. (2021). Aberrant Intraregional Brain Activity and Functional Connectivity in Patients With Diarrhea-Predominant Irritable Bowel Syndrome. *Front Neurosci* 15**,** 721822. doi: 10.3389/fnins.2021.721822.

Gupta, A., Kilpatrick, L., Labus, J., Tillisch, K., Braun, A., Hong, J.Y., et al. (2014). Early adverse life events and resting state neural networks in patients with chronic abdominal pain: evidence for sex differences. *Psychosom Med* 76(6)**,** 404-412. doi: 10.1097/psy.0000000000000089.

Hubbard, C.S., Becerra, L., Heinz, N., Ludwick, A., Rasooly, T., Wu, R., et al. (2016). Abdominal Pain, the Adolescent and Altered Brain Structure and Function. *PLoS One* 11(5)**,** e0156545. doi: 10.1371/journal.pone.0156545.

Icenhour, A., Witt, S.T., Elsenbruch, S., Lowén, M., Engström, M., Tillisch, K., et al. (2017). Brain functional connectivity is associated with visceral sensitivity in women with Irritable Bowel Syndrome. *Neuroimage Clin* 15**,** 449-457. doi: 10.1016/j.nicl.2017.06.001.

Longarzo, M., Quarantelli, M., Aiello, M., Romano, M., Del Prete, A., Cimminiello, C., et al. (2017). The influence of interoceptive awareness on functional connectivity in patients with irritable bowel syndrome. *Brain Imaging Behav* 11(4)**,** 1117-1128. doi: 10.1007/s11682-016-9595-5.
